# Supplementary material for: IL-33 Induces an Antiviral Signature in Mast Cells but Enhances Their Permissiveness for Human Rhinovirus Infection
Source: Viruses. 2022 Nov 1;14(11):2430. doi: 10.3390/v14112430 (PMC9699625; doi:10.3390/v14112430)

viruses

MDPI

**Materials and Methods**

*2.1. Mast Cell Culture*

The human MC line, LAD2, was obtained from Dr. A. Kirshenbaum (National Institutes of Health, Bethesda, USA) [43] and was cultured in Stem Pro-34® serum-free basal medium (Gibco, ThermoFisher, Paisley, UK) supplemented with 2-mM L-glutamine, 100 IU/mL penicillin, 100 µg/mL streptomycin, and 100 ng/mL recombinant stem cell factor (SCF) (Peprotech, London, UK), as previously described [23]. Primary cord blood-derived MCs (CBMCs) were generated as previously described [23] from a commercial source of CD34+ cord blood progenitor cells (StemCell technologies, Grenoble, France). Briefly, CD34+ cells were cultured in StemPro® medium supplemented with 2 mM L-glutamine, 100 IU/mL penicillin, 100 µg/mL streptomycin, IL-3 (30 ng/mL, 1 week only, Peprotech, UK), IL-6 (100 ng/mL, Peprotech, London, UK) and SCF (100 ng/mL) for 3 weeks by hemidepletion before culture media was completely replaced weekly thereafter. After 8-10 weeks, CBMCs were assessed for purity by FcεRI and CD117 expression by flow cytometry

*2.2. Rhinovirus Stocks*

Human rhinovirus serotype 16 (HRV16, major group, HRV-B species) stocks were generated using H1-HeLa cells obtained from ATCC as previously described [23]. Virus titres of cell-free supernatant stocks were determined by tissue culture infective dose 50% ($TCID_{50}$)/mL according to the Spearman-Karber method. Controls of UV-irradiated HRV16 (1,200 mJ/$cm^2$ on ice for 50 min) were included in all experiments.

*2.3. Cell treatments and Infections*

Human MCs (1 × $10^6$/mL) were incubated with IL-33 (1–10 ng/mL, R&D Systems, Abingdon, UK) for 6–24 h in a humidified 37°C incubator with 5% $CO_2$ before transcriptomic and qPCR analysis. For RV16 infection, the LAD2 cell line or CBMCs were incubated with increasing MOI (1–7.5) of infectious virus or UV-irradiated virus (as a control) for 1 h whilst rocking in the dark at 36 opm. Cells were then washed twice to remove unbound virus, resuspended in StemPro media (0.5–1 × $10^6$/mL) and incubated for 24 h in a humidified 37°C incubator with 5% $CO_2$ before harvesting. For ICAM-1 blocking experiments, LAD2 MCs or CBMCs (1 × $10^6$/mL) were treated with 10 ng/mL IL-33 for 23 h followed by 1 h with mouse anti-human ICAM-1 (clone BBIG-I1 (11C81), 10 µg/mL, R&D Systems, Abingdon, UK) or IgG2a isotype control (10 µg/mL, R&D Systems, Abingdon, UK). Following HRV16 infection of MCs, the antibodies were re-introduced and cultures incubated for a further 24 h. Experimental layouts are shown below (Figure B1).

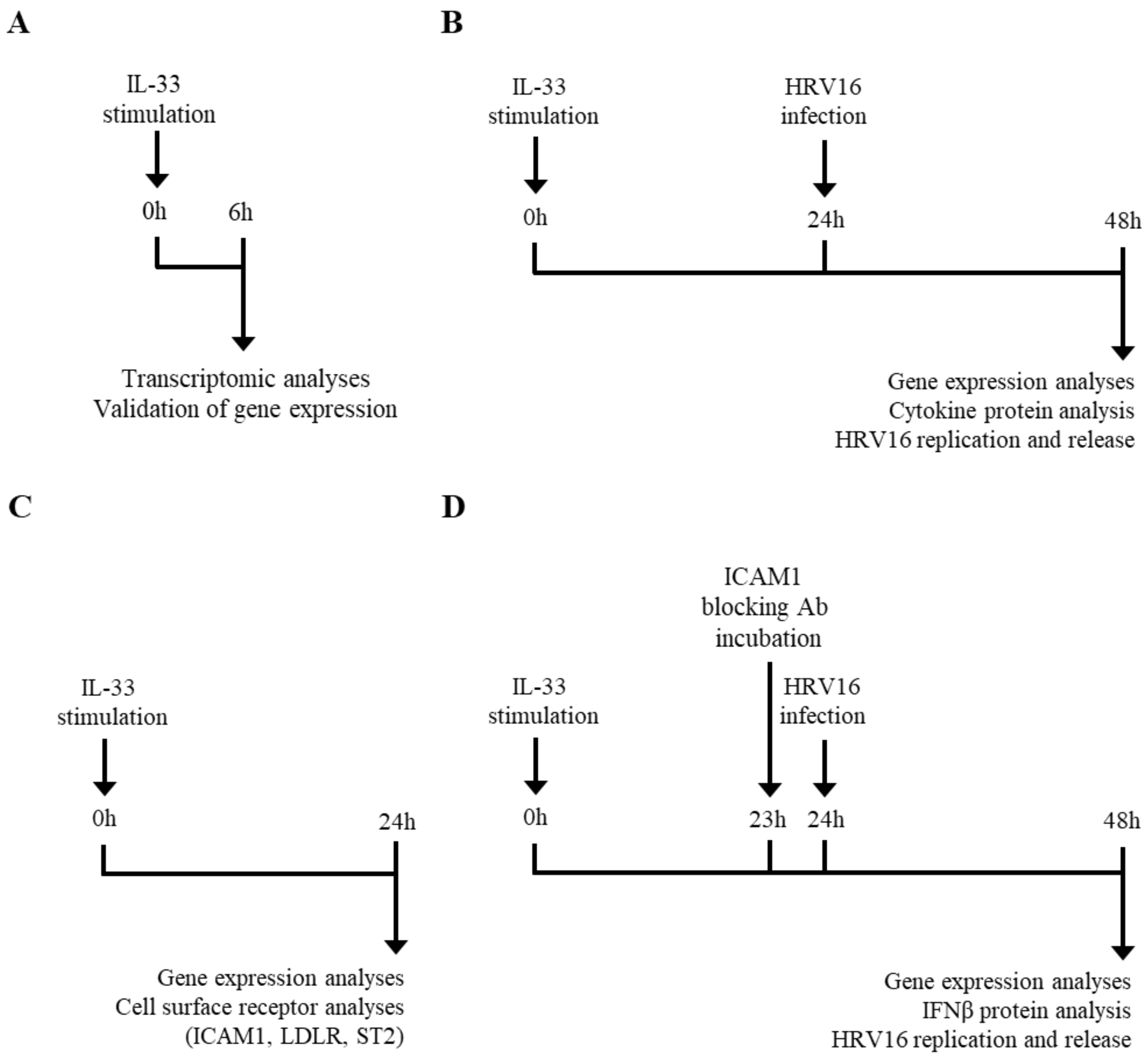


**Figure S1.** Experimental layout of stimulations of MCs with IL-33 and HRV16. LAD2 MCs were treated for 6 h with IL-33 (10 ng/mL) prior to transcriptomic analyses (**A**, Figures 1-2, S2, Table S1) whereas LAD2 or CBMCs were treated with IL-33 for 24 h prior to HRV16 infection and samples collected 24 h later for gene expression, IFN-beta protein release, HRV16 replication and release (**B**, Figures 3-4, 6A, A2-A3). For cell surface receptor analyses by RT-qPCR and flow cytometry (ICAM1, LDLR, ST2), LAD2 MCs or CBMCs were stimulated with IL-33 (10 ng/mL) for 24 h prior to analyses (**C**, Figure 5A-B, 6B-C, A4-A5). Where an ICAM1 blocking antibody was used, the blocking antibody (Ab) was added to LAD2 MC or CBMCs 23h post-IL-33 stimulation for 1 h before infection with HRV16. Samples were then collected 24 h post-HRV16 infection (**D**, Figure 5C-E, 6D-F).

*2.4. mRNA Extraction, RNA Sequencing and Transcriptomic Analysis*

The LAD2 MC line was incubated with IL-33 (10 ng/mL) or media control for 6 h in StemPro® media containing SCF (100 ng/mL). Total RNA was isolated using either the Trizol extraction method as previously described [23] or using commercially available kits (RNeasy minikit, Qiagen (Manchester, UK) or Monarch total RNA miniprep kit (New England Biolabs, Hitchin, UK) and quantified for RNA quantity and quality (Nanodrop, ThermoFisher, Paisley, UK). Next generation sequencing was performed by Novogene (Cambridge, UK) at a read depth of 20 million reads per sample following quality control checks and data pre-processing. Raw reads were stored as FASTQ files, uploaded to the Iridis computer cluster (University of Southampton, Southampton, UK), mapped to the human genome (HISAT2), annotated with gene names and converted to counts (SAMtools). Using RStudio, the count matrix was then adjusted for batch effects (ComBat Seq within sva package) before low counts were filtered out (EdgER) and those remaining were normalised using a weighted trimmed mean of the log expression ratios (trimmed mean of M values (TMM)). The resultant expression matrix was used to create Multidimensional scaling plots (limma, Rstudio) and Heatmaps (heatmap.2, RStudio) and a fitted to a generalised linear model (quasi-likelihood F-test) for differential expression. Volcano plots were generated of all detected genes (EnhancedVolcano package, RStudio). Differentially expressed genes (DEGs) were defined as genes with a $log_2$(fold change) ($log_2$FC)>1.5 and a False Discovery Rate (FDR)-adjusted p value <0.05. The significantly upregulated genes were inputted into g:profiler (https://biit.cs.ut.ee/gprofiler/gost) with the

background gene set to the total human genome and term size limited (5 -10,000) for Gene ontology (GO) and pathways analysis (KEGG). The resulting GO terms were then submitted to REVIGO for visualisation and gene set enrichment analysis (GSEA). Data are available at GSE2162692.*5. RT-qPCR*

Isolated RNA was DNAse I treated (Stemcell Technologies, Cambridge, UK), reverse transcribed to cDNA using precision nanoScript2 reverse transcription kit (PrimerDesign, Chandlers Ford, UK) and 12.5 ng used as a template in quantitative PCR (qPCR) with Precision Plus double dye primers for housekeeping genes (HKGs; *GAPDH*, *UBC*) or genes of interest (*IFIH1, IRF1, TNFA, IFNB1, IFNL1, MDA5, OAS1, CXCL10, IL6, CCL5*) or SYBR® green primers for genes of interest (*ICAM1*) used to quantify amplification of genes. All reactions were performed in duplicate for 50 cycles and gene expression analysed using a real-time PCR iCycler (BioRad, Hemel Hempstead, UK). For SYBR green detection-based reactions, melt curves were performed to ensure single PCR product formation. Gene expression was normalised to the geometric means of HKGs and fold changes in gene expression calculated relative to UV-HRV16 controls according to the $^{\Delta\Delta}$Ct method and expressed as $2^{-\Delta\Delta Ct}$. Viral RNA copy number was determined against a standard curve of known copies of HRV16 (Primerdesign, Chandlers Ford, UK).

### *2.5. Flow Cytometry*

Human MCs were washed with FACS buffer (PBS + 0.5% (v/v) BSA + 2 mM EDTA) prior to blocking with block buffer (PBS + 10% (v/v) heat inactivated human serum + 2 mM EDTA) for 20 min on ice. Cells ($0.1\times10^6$/100 µL) were then incubated with fluorescently labelled antibodies, FITC-conjugated mouse anti-human ICAM1 (clone RR1/1), subclass $IgG_1$) or mouse $IgG_1$ isotype control (eBiosciences, Cheshire, UK) for 30 min with the addition of eBioscience™ Fixable Viability Dye eFluor™ 660 (Thermo Fisher Scientific, Paisley, UK) on ice prior to washing and resuspending in 300 µL FACS buffer. At least 10,000 events were collected for analysis and gating was performed on non-APC expressing cells, i.e., live cells, and ICAM1 geometric means expressed minus that of isotype controls. Flow cytometry was performed using a BD FACSCalibur flow cytometer (BD Biosciences, Oxford, UK) and data analysed using FlowJo software (version 7.6.5, BD, Oregon, USA).

### *2.6. $TCID_{50}$ Assay*

The number of infectious virus particles in cell-free supernatants was determined by the $TCID_{50}$ assay where a 10-fold serial dilution of supernatants in quadruplicate were added to OHIO HeLa cells ($0.2\times10^6$/well, 96-well plate). After 96 h, cytopathic effect (CPE) was visualised by staining monolayers with crystal violet solution (0.13% (w/v), 1.825% (v/v) formaldehyde, 5% ethanol (v/v), 90% PBS (v/v) for 30 min in the dark. Excess crystal violet was removed by gentle rinsing and the number of wells where at least 50% of the monolayer had been lysed (i.e., 50% CPE) was used to calculate $TCID_{50}$/mL using the Spearman-Karber Method

### *2.7. Statistical Analysis*

Paired non-parametric data were analysed by Wilcoxon signed rank test for matched pair comparisons. Un-paired non-parametric data were analysed by Kruskal–Wallis one-way ANOVA with Dunn's correction for multiple comparisons or Mann–Whitney ranked sum test and normalised data were analysed by Student's t–test. All data were analysed using GraphPad Prism (GraphPad Software, Inc., San Diego, CA, USA)

## Tables and Figures

**Table S1.** Top 50 upregulated and downregulated genes in IL-33-activated MCs compared to control samples. Genes were ranked by fold change and the top 50 upregulated and downregulated genes were taken. Genes in bold were antiviral genes identified through KEGG pathway analysis.

| Top 50 upregulated genes in C vs C+IL33 by FC | | | | | Top 50 downregulated genes in C vs C+IL33 by FC | | | | |
|---|---|---|---|---|---|---|---|---|---|
| | Gene | FDR | FC | $log_2FC$ | | Gene | FDR | FC | $log_2FC$ |
| 1 | *LINC02605* | 1.82E-14 | 295.50 | 8.207 | 1 | *PLCH1* | 7.86E-15 | -10.07 | -3.33 |
| 2 | ***EBI3*** | **8.94E-24** | **287.02** | **8.165** | 2 | *MAP2K6* | 2.30E-13 | -6.21 | -2.63 |
| 3 | *FAM131C* | 1.54E-05 | 230.56 | 7.849 | 3 | *ARHGEF4* | 8.44E-06 | -5.63 | -2.49 |
| 4 | *CXCL1* | 4.54E-06 | 179.02 | 7.484 | 4 | *TBXA2R* | 0.001059 | -4.84 | -2.28 |
| 5 | *MIR4422HG* | 7.02E-09 | 157.26 | 7.297 | 5 | *HBQ1* | 2.57E-05 | -4.75 | -2.25 |
| 6 | *PRRX1* | 0.000152 | 150.44 | 7.233 | 6 | *SCN2A* | 4.85E-05 | -4.73 | -2.24 |

| | | | | |
|---|---|---|---|---|
| 7 | *IL13* | 2.00E-18 | 134.27 | 7.069 |
| 8 | *AL353608.3* | 0.000397 | 94.03 | 6.555 |
| 9 | *AC007336.2* | 4.08E-06 | 82.54 | 6.367 |
| 10 | *TNF* | 9.71E-16 | 80.34 | 6.328 |
| 11 | *C3* | 1.19E-18 | 67.23 | 6.071 |
| 12 | *AC083837.1* | 2.28E-19 | 62.90 | 5.975 |
| 13 | *MFAP5* | 2.71E-05 | 60.59 | 5.921 |
| 14 | *CSF3* | 0.003747 | 59.26 | 5.889 |
| 15 | *TNFAIP2* | 3.13E-21 | 56.45 | 5.819 |
| 16 | *ITGA1* | 1.38E-18 | 55.33 | 5.79 |
| 17 | *BCL2A1* | 5.41E-16 | 46.11 | 5.527 |
| 18 | *SERPINE1* | 1.74E-14 | 38.67 | 5.273 |
| 19 | *LRFN5* | 4.34E-12 | 37.37 | 5.224 |
| 20 | *CXCL10* | 1.79E-06 | 35.31 | 5.142 |
| 21 | *NINJ1* | 1.73E-23 | 34.94 | 5.127 |
| 22 | *PDZD2* | 1.35E-20 | 32.72 | 5.032 |
| 23 | *SECTM1* | 1.01E-12 | 30.25 | 4.919 |
| 24 | *TSPAN18* | 7.29E-11 | 29.10 | 4.863 |
| 25 | *CXCL8* | 9.57E-14 | 29.02 | 4.859 |
| 26 | *TMEM132E* | 1.76E-09 | 28.76 | 4.846 |
| 27 | *TRABD2B* | 3.02E-09 | 28.74 | 4.845 |
| 28 | *PTGIR* | 5.22E-13 | 28.01 | 4.808 |
| 29 | *GBP5* | 4.11E-14 | 26.87 | 4.748 |
| 30 | *ACAN* | 5.12E-08 | 26.21 | 4.712 |
| 31 | *ROR1-AS1* | 5.11E-14 | 24.92 | 4.639 |
| 32 | *NFKBIA* | 6.54E-17 | 22.16 | 4.47 |
| 33 | *IL5* | 1.67E-07 | 21.92 | 4.454 |
| 34 | *PTP4A3* | 2.09E-14 | 21.60 | 4.433 |
| 35 | *AC010247.1* | 6.07E-16 | 21.53 | 4.428 |
| 36 | *N4BP3* | 1.53E-15 | 21.21 | 4.407 |
| 37 | *AC083864.5* | 1.45E-10 | 20.84 | 4.381 |
| 38 | *IL1B* | 9.34E-07 | 20.72 | 4.373 |
| 39 | *TNFRSF9* | 1.73E-23 | 20.72 | 4.373 |
| 40 | *GFPT2* | 1.37E-14 | 20.72 | 4.373 |
| 41 | ***TLR7*** | **3.83E-10** | **20.14** | **4.332** |
| 42 | *CXCL3* | 5.07E-11 | 20.06 | 4.326 |
| 43 | *CCL3L3* | 6.88E-18 | 19.64 | 4.296 |
| 44 | *VSTM2A* | 5.12E-07 | 19.20 | 4.263 |
| 45 | *TRAF1* | 4.70E-27 | 18.97 | 4.246 |
| 46 | *MMP10* | 3.04E-07 | 18.83 | 4.235 |
| 47 | *IL2RA* | 8.18E-17 | 18.58 | 4.216 |
| 48 | *POU2F2* | 4.64E-27 | 18.46 | 4.206 |
| 49 | *LINC01215* | 7.97E-17 | 18.46 | 4.206 |
| 50 | *TSLP* | 0.001398 | 17.68 | 4.144 |

| | | | | |
|---|---|---|---|---|
| 7 | *TMEM38A* | 8.94E-07 | -4.47 | -2.16 |
| 8 | *ADRA2A* | 2.01E-10 | -4.29 | -2.10 |
| 9 | *AC019197.1* | 0.002786 | -4.23 | -2.08 |
| 10 | *PLXNA4* | 1.42E-09 | -4.18 | -2.06 |
| 11 | *ANGPT2* | 2.91E-15 | -4.16 | -2.06 |
| 12 | *SCN3A* | 2.40E-08 | -4.16 | -2.06 |
| 13 | *ZBTB16* | 1.43E-08 | -4.09 | -2.03 |
| 14 | *GDF2* | 9.69E-14 | -4.08 | -2.03 |
| 15 | *SHE* | 0.000257 | -4.01 | -2.00 |
| 16 | *RSPO2* | 4.44E-07 | -3.96 | -1.99 |
| 17 | *NFE2* | 5.33E-13 | -3.87 | -1.95 |
| 18 | *GIPC3* | 0.031608 | -3.74 | -1.90 |
| 19 | *COBLL1* | 1.38E-09 | -3.67 | -1.88 |
| 20 | *PTPN5* | 1.19E-18 | -3.68 | -1.88 |
| 21 | *AC092155.1* | 0.002428 | -3.65 | -1.87 |
| 22 | *AL670729.3* | 4.71E-07 | -3.63 | -1.86 |
| 23 | *LINC01366* | 4.59E-12 | -3.63 | -1.86 |
| 24 | *CTPS2* | 4.16E-05 | -3.63 | -1.86 |
| 25 | *DACH1* | 7.09E-09 | -3.58 | -1.84 |
| 26 | *FEV* | 0.114045 | -3.59 | -1.84 |
| 27 | *DMTN* | 1.26E-05 | -3.55 | -1.83 |
| 28 | *UNC80* | 1.88E-07 | -3.55 | -1.83 |
| 29 | *CXXC4* | 1.32E-05 | -3.53 | -1.82 |
| 30 | *PTGDR2* | 6.14E-06 | -3.51 | -1.81 |
| 31 | *SEC14L5* | 1.00E-12 | -3.40 | -1.76 |
| 32 | *CEBPA* | 2.50E-05 | -3.40 | -1.77 |
| 33 | *NPY2R* | 1.38E-09 | -3.34 | -1.74 |
| 34 | *EDNRB* | 4.65E-11 | -3.33 | -1.74 |
| 35 | *MEX3B* | 1.15E-14 | -3.31 | -1.73 |
| 36 | *CEBPE* | 4.12E-08 | -3.27 | -1.71 |
| 37 | *RD3* | 5.62E-15 | -3.27 | -1.71 |
| 38 | *AC097713.1* | 0.007003 | -3.27 | -1.71 |
| 39 | *A4GALT* | 5.74E-05 | -3.22 | -1.69 |
| 40 | *GXYLT1P6* | 6.35E-08 | -3.18 | -1.67 |
| 41 | *PTCH1* | 5.85E-11 | -3.16 | -1.66 |
| 42 | *HEMGN* | 0.014634 | -3.16 | -1.66 |
| 43 | *JPH4* | 1.37E-16 | -3.14 | -1.65 |
| 44 | *CLK3P2* | 2.61E-08 | -3.12 | -1.64 |
| 45 | *PADI2* | 3.17E-10 | -3.08 | -1.63 |
| 46 | *CNNM1* | 0.028685 | -3.09 | -1.63 |
| 47 | *GPAT3* | 1.25E-12 | -3.08 | -1.62 |
| 48 | *RAB6C-AS1* | 0.000126 | -3.06 | -1.62 |
| 49 | *TOGARAM2* | 1.27E-05 | -3.07 | -1.62 |
| 50 | *CRISPLD2* | 0.000138 | -3.00 | -1.58 |

**Figure S2.** Violin plots of antiviral genes associated with HRV infection in LAD2 MCs. From the transcriptomic data the significant DEGs associated with HRV infection of BECs were identified in the LAD2 MC + IL-33 (10 ng/mL) dataset and replicate samples displayed as violin plots for control (green) and IL-33 (blue) treated samples, *n*=7.

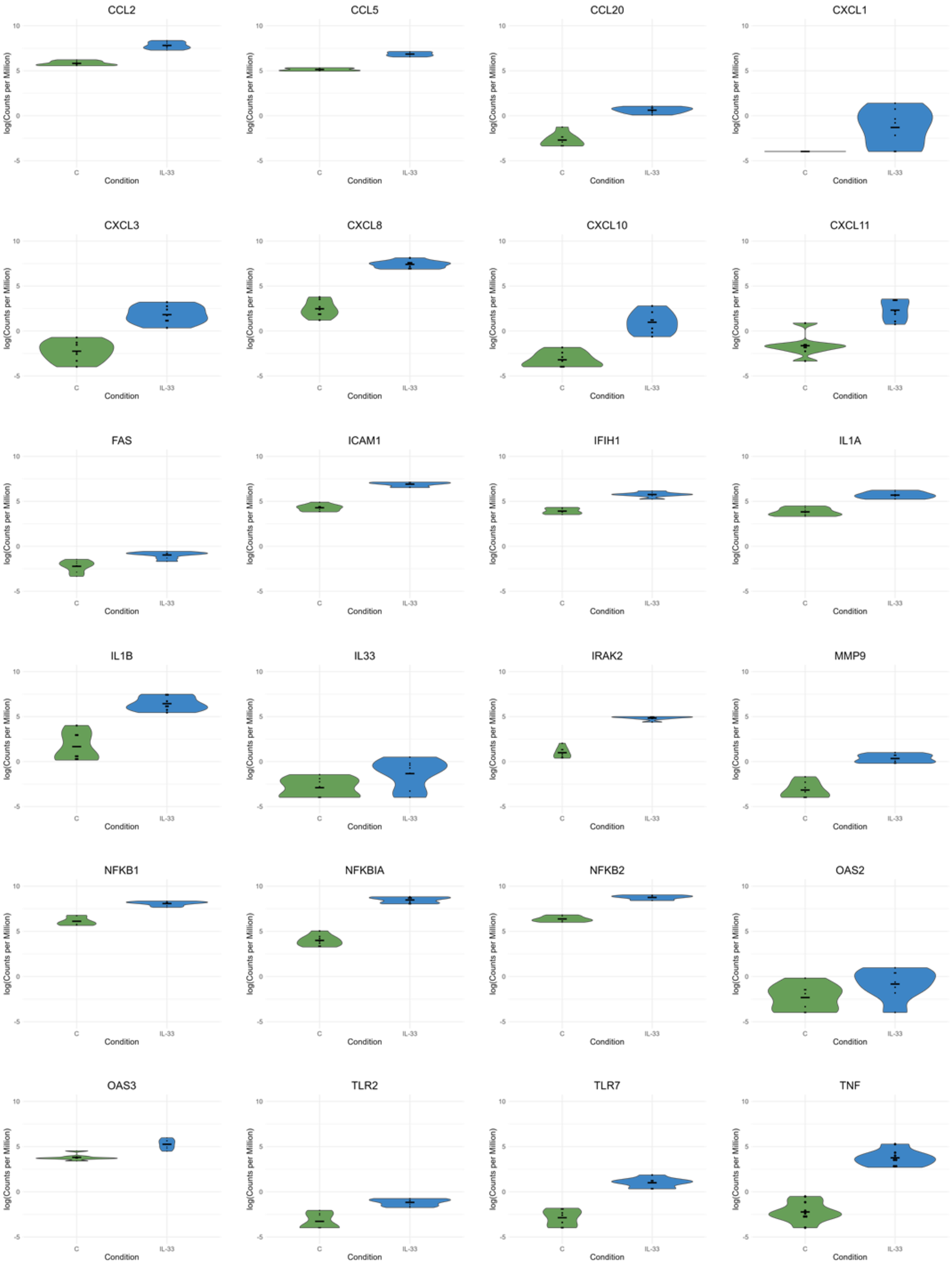

**Figure S3.** Concentration-dependent induction of IFN and IFN-inducible genes by IL-33 in MCs. Validation of *IFNB1*, *IFNL1*, *IFIH1*, *OAS1*, *CCL5* and *CXCL10* gene induction by IL-33 (1, 5, 10 ng/mL) treatment for 24 h, *n*=5.

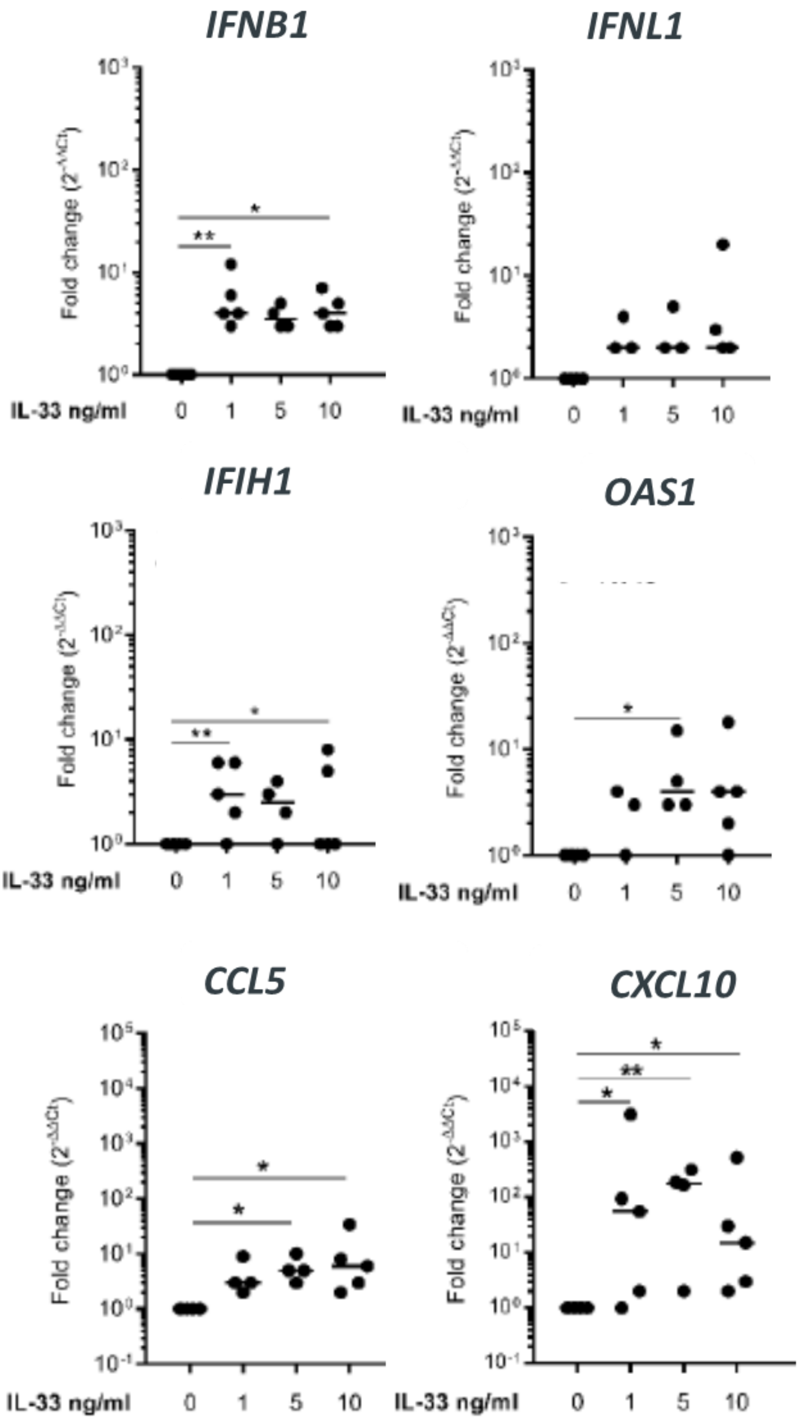

**Figure S4:** HRV16 affects IL-33-dependent CXCL10 and IL6 release. mRNA expression of *CXCL10* (**A**) and *IL6* (**B**) and IL6 protein (**C**) in LAD2 MCs pretreated with or without IL-33 (1–10 ng/mL) for 24 h prior to HRV16 or UV-HRV16 (control) infection (MOI 7.5) for a further 24 h, *n*=5-6.

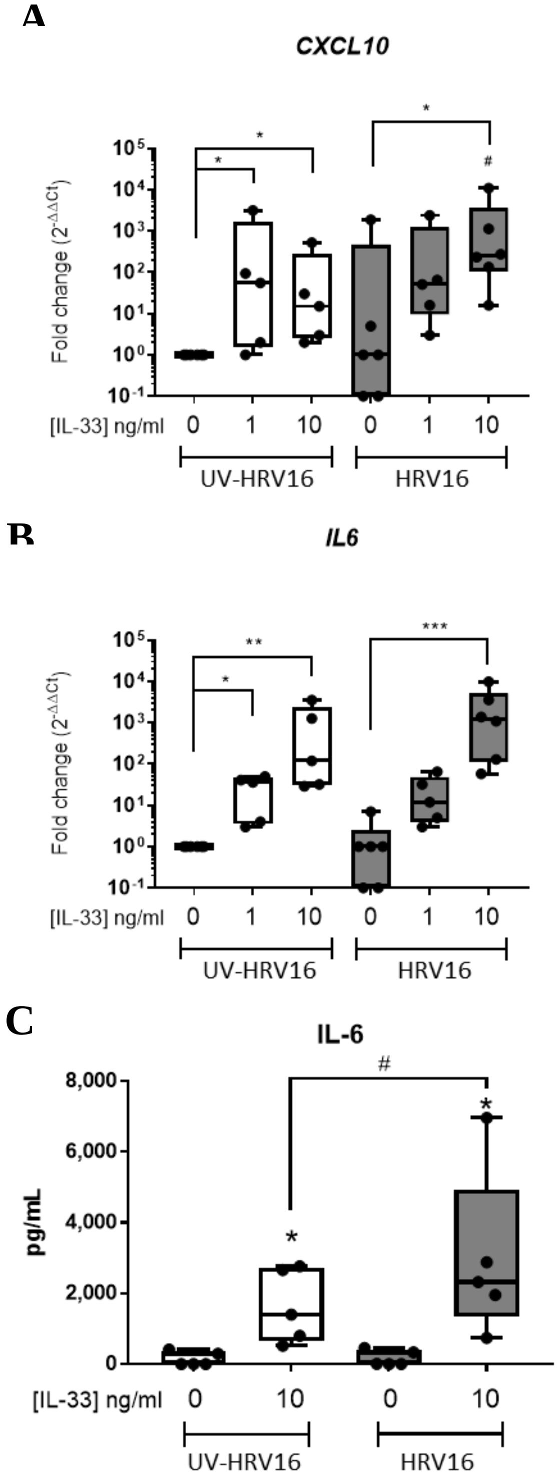

**Figure S5:** IL-33 enhances ICAM1, LDLR but not CDHR3. *ICAM1* (A) and *CDHR3* (B) gene expression in MCs treated with IL-33 (10 ng/mL) for 24 h, *n*=6. C, LDLR cell surface expression in MCs treated with IL-33 (10 ng/mL) for 24 h, *n*=4-7.

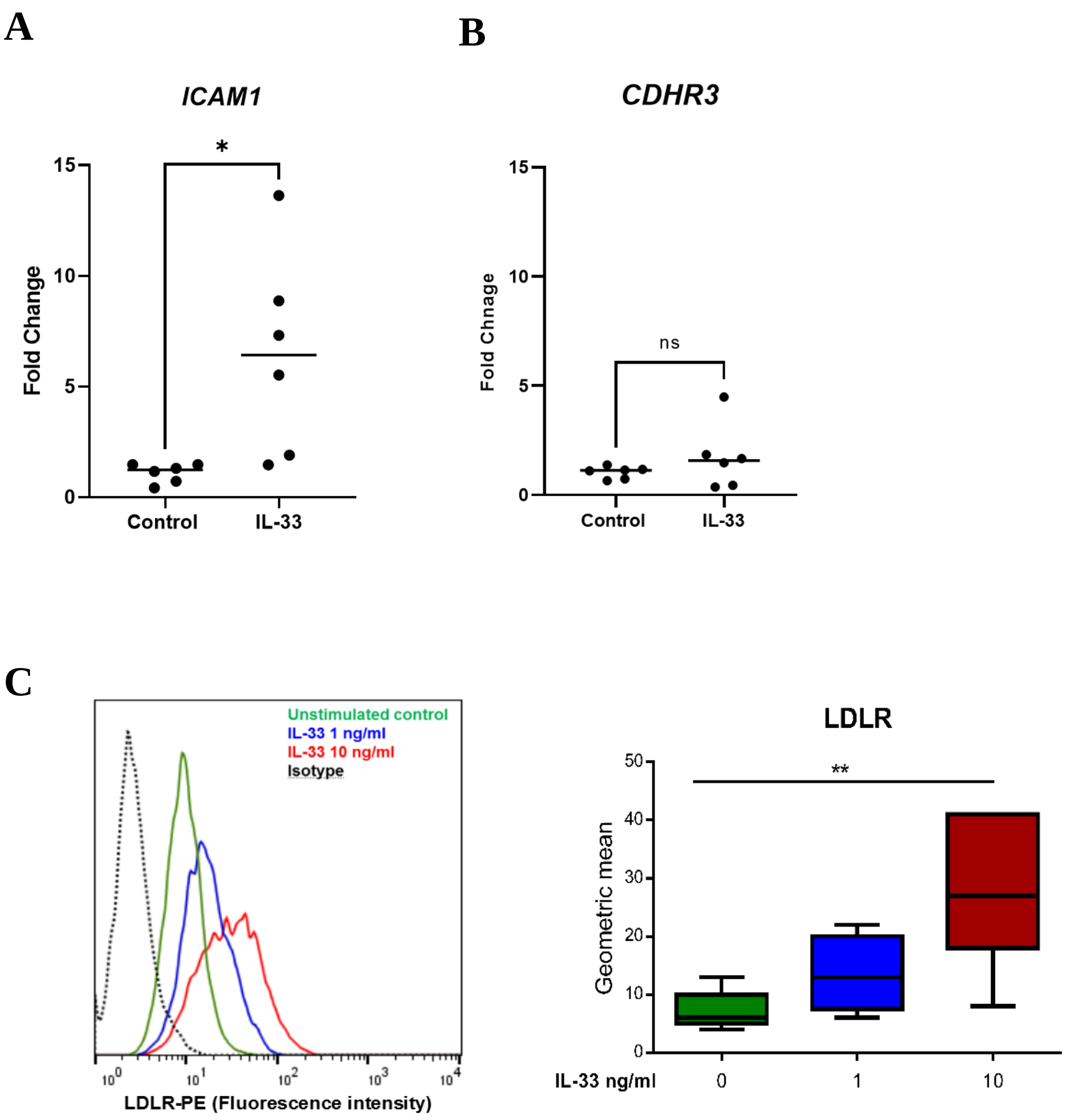

**Figure S6:** IL-33 enhances membrane ST2 in LAD2 MCs and CBMCs. **A**, ST2 cell surface expression in LAD2 MCs treated with IL-33 (10 ng/mL) for 24h, n=8. **B**, ST2 cell surface expression in CBMCs treated with IL-33 (10 ng/mL) for 24 h, *n*=2.

**A**

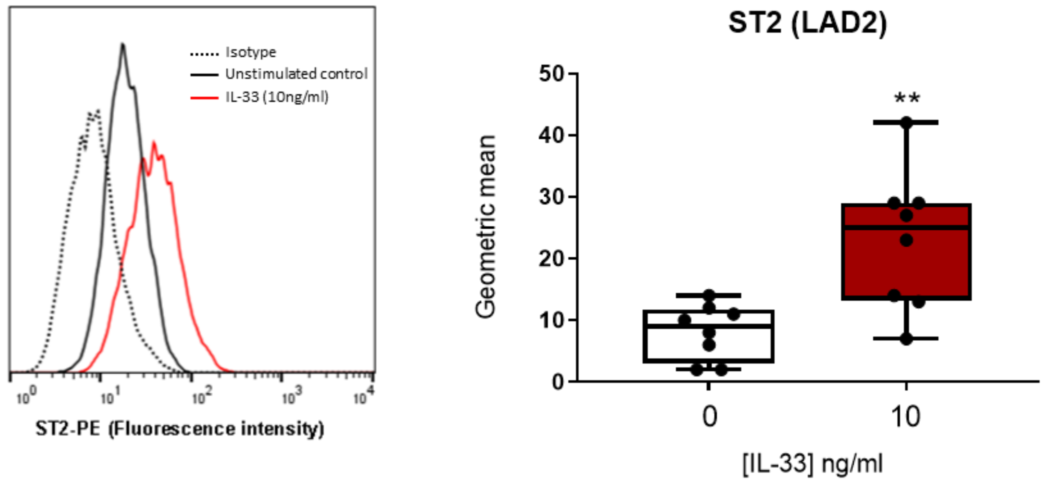


**B**

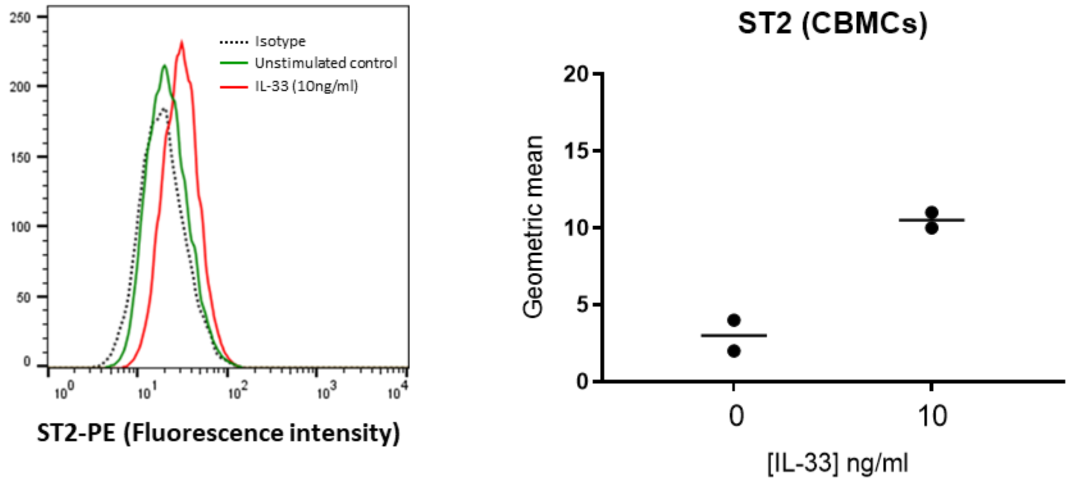

Supplement: Supplementary file 1 [file viruses-14-02430-s001.zip › viruses-1968628-supplementary.pdf]
